# Selective episiotomy vs. implementation of a non-episiotomy protocol: a randomized clinical trial

**DOI:** 10.1186/s12978-017-0315-4

**Published:** 2017-04-24

**Authors:** M. M. Amorim, Isabela Cristina Coutinho, Inês Melo, Leila Katz

**Affiliations:** 1 Instituto de Medicina Integral Prof. Fernando Figueira, Women’s Healthcare Centre, 50070-550, Recife, Pernambuco Brazil; 2 Instituto Paraibano de Pesquisa Professor Joaquim Amorim Neto, Rua Neusa Borborema, 300, Santo Antônio, Campina Grande, 58406-120 Brazil

**Keywords:** Episiotomy, Vaginal delivery, Perineum, Randomized controlled trial

## Abstract

**Background:**

Despite all the evidence corroborating the selective use of episiotomy and although routine use of the procedure is contraindicated, there are no evidences corroborating if episiotomy is necessary in any circumstance. The present clinical randomized trial was performed to compare maternal and perinatal outcomes in women submitted to a non-episiotomy protocol versus one of selective episiotomy.

**Methods:**

An open-labelled, randomized clinical trial was carried out in a tertiary teaching hospital in Recife, Northeastern Brazil. Women in labor with a full-term live foetus, dilatation of 6 to 8 cm and cephalic presentation (vertex position) were included. Exclusion criteria consisted of bleeding disorders and an indication for a caesarean section. After signing the consent form, 241 women were randomized to a non-episiotomy protocol (the experimental group) or to a selective episiotomy group (the control group). No episiotomies were to be performed in the experimental group except under exceptional circumstances. In the control group, selective episiotomies were to be performed in accordance with the healthcare professionals’ clinical judgement. Maternal and perinatal outcomes were evaluated. Ratio Risk (RR) and the 95% confidence interval (95% CI) were calculated for our outcomes.

**Results:**

The analysis include 115 women assigned to a non-episiotomy protocol and 122 to selective episiotomy. There was no difference between the two groups with respect to maternal or perinatal outcomes. The episiotomy rate was similar (two cases in each group, about 1.7%), as was the duration of the second stage of labor, the frequency of perineal tears, severe perineal trauma, need for perineal suturing and blood loss at delivery.

**Conclusions:**

A non-episiotomy protocol appears to be safe for mother and child, and highlights the need to investigate whether there is, in fact, any indication for this procedure.

**Trial registration:**

This trial was registered at ClinicalTrials.gov under reference number (NCT02178111).

## Plain english summary

Despite all the evidence corroborating the selective use of episiotomy and although routine use of the procedure is contraindicated, there are no evidences corroborating if episiotomy is necessary in any circumstance. The present clinical randomized trial was performed to compare maternal and perinatal outcomes in women submitted to a non-episiotomy protocol versus one of selective episiotomy. After signing the consent form, 241 women were randomized to a non-episiotomy protocol (the experimental group) or to a selective episiotomy group (the control group). No episiotomies were to be performed in the experimental group except under exceptional circumstances. In the control group, selective episiotomies were to be performed in accordance with the healthcare professionals’ clinical judgement. Of the 241 women enrolled, four were excluded because of a post-randomization indication for a caesarean section. Of the remaining 237, 115 were assigned to a non-episiotomy protocol and 122 to selective episiotomy. There was no difference between the two groups with respect to maternal or perinatal outcomes. The episiotomy rate was similar with two episiotomies in each group (1.7%), as was the duration of the second stage of labor, the frequency of perineal tears, severe perineal trauma, need for perineal suturing and blood loss at delivery. In conclusion, a non-episiotomy protocol appears to be safe for mother and child, and highlights the need to investigate whether there is, in fact, any indication for this procedure.

## Background

Despite all the evidence corroborating the selective use of episiotomy and although routine use of the procedure is contraindicated, the actual indications for episiotomy in modern obstetric practice still remain to be clarified [1]. According to the American College of Obstetricians and Gynecologists (ACOG), “*based on the existent evidence, there are no specific situation in which episiotomy is essential, and the decision to perform an episiotomy should be based on clinical considerations*” [2]. The World Health Organization (WHO) recommends an episiotomy rate of 10% as “*a good goal to pursue*” [3], based on the previous results of a randomized clinical trial conducted in the United Kingdom and published in 1984 [4].

A Cochrane systematic review raises the question of what indeed are the real indications for episiotomy. Suggested answers have included premature delivery, breech presentation, fetal macrosomia, shoulder dystocia, instrumental delivery (forceps or vacuum extraction), non-reassuring fetal heart rate, and rigid perineum or imminent perineal tears [5]. It has been debated, however, whether these situations do indeed represent indications for episiotomy and clearly this question merits further investigation in randomized clinical trials. Although it is obvious that routine episiotomy should be avoided, there is no solid evidence corroborating any indication whatsoever for episiotomy. The benefits of the use of the procedure in selected situations remain controversial, including in instrumental deliveries [6]. In an article proposing that the routine episiotomy era has come to an end, Scott suggests that the natural forces of labor should be allowed to gradually distend the perineum and recalls the adage already quoted by Eason and Feldman 15 years ago: “*Don’t just do something, sit there*!” [7, 8]. Recently, some authors suggested that episiotomy should never be performed [1, 9]. The question, however, has not yet been adequately evaluated in randomized clinical trials.

A review of the Medline, Lilacs/SciELO, EMBASE and Scopus databases and of the Cochrane Library using the keywords “*episiotomy*” and “*vaginal delivery*” in Portuguese, English and Spanish, with the filters “*randomized clinical trials*” and “*systematic review*”, failed to reveal any randomized clinical trials on the subject of not performing episiotomy during vaginal delivery. All the trials published in the literature compare selective versus routine episiotomy and they were not designed to assess the need for episiotomy in any clinical setting [5]. This is an inherent limitation for its interpretation.

The present clinical trial was performed to compare maternal and perinatal outcomes in women submitted to a non-episiotomy protocol versus one of selective episiotomy.

## Methods

This study was conducted at the Women’s Healthcare Centre in the *Instituto de Medicina Integral Prof. Fernando Figueira* (IMIP), Recife, Brazil, between July and September 2014. The protocol is registered at ClinicalTrials.gov under number NCT02178111 and was published in *Reproductive Health* [1].

This is an open, randomized clinical trial conducted with clinically stable women in active labor with a live, full-term foetus (37 to 41 weeks of pregnancy) in cephalic presentation (vertex position), and with dilatation of 6 to 8 cm. Exclusion criteria consisted of pregnancy bleeding disorders (premature detachment of the placenta); indication for a caesarean section; cephalopelvic disproportion, non-reassuring fetal heart rate, dystocia; women incapable of giving their consent and women with no responsible accompanying person. In addition, women who were submitted to a caesarean section after enrolment to the study were excluded (post-randomization exclusion).

Sample size was calculated using the Open Epi software program, version 2.3 (Atlanta, GA, USA). Predicting an episiotomy rate of 1% in the non-episiotomy group versus 10% in the selective episiotomy group [3, 4], for a power of 80% and a 95% confidence level, 200 women would need to be enrolled. This number was increased by 20% to compensate for any possible losses, resulting in an estimated sample size of 240 women. A list of random numbers was generated using the Random Allocation software program, version 1.0. To compensate for any post-randomization losses, the list consisted of 250 random numbers. Opaque, sealed, consecutively numbered envelopes containing each participant’s allocation were then prepared. Each allocation envelope was only opened during the second stage, defined as when dilatation is complete, the head of the foetus is fully engaged in the pelvis and there is a spontaneous urge to push. Allocation concealment was thus assured up to this moment.

For the experimental group, the healthcare professionals were instructed to base their management on the principle that episiotomy is unnecessary even in situations in which the literature suggests that it may confer some benefit. Therefore, no episiotomy was to be performed in this group except under exceptional circumstances, i.e. those in which clinical judgement would deem the procedure absolutely necessary. For the control group, the healthcare professionals should perform episiotomy selectively according to their clinical judgement, i.e. in line with the routine procedure at the institute.

The primary maternal outcomes were episiotomy rate; duration of second stage; frequency of spontaneous lacerations (degree of the laceration and the location: anterior or posterior); frequency of instrumental delivery; frequency of perineal trauma (any type: episiotomy or tearing); postpartum blood loss; need for perineal suturing, and the number of suture threads. The primary perinatal outcomes were 1^st^ and 5^th^ minute Apgar scores; need for neonatal resuscitation and umbilical cord blood pH. The secondary maternal outcomes evaluated were frequency of severe perineal trauma; complications with perineal suturing (edema, ecchymosis, hyperemia, secretion, hematoma, dehiscence, perineal pain and infection) identified during postnatal consultation; intensity of postpartum perineal pain assessed according to a visual pain scale [10, 11], and maternal satisfaction. The secondary perinatal outcomes were neonatal morbidity (neonatal asphyxia and the duration of neonatal hospitalization) and admission of the newborn infant to the neonatal ICU. In the cases in which episiotomy was performed, the indications for the procedure and the characteristics of the women submitted to it were also analyzed.

The women were accompanied during delivery according to a humanized childbirth care model based on evidence and on the WHO recommendations [3]. This model favors non-supine positions, non-pharmacological pain relief methods, continuous support during delivery and techniques for protecting the perineum, all in accordance with the woman’s preferences and the routine at the institute.

The duration of second stage of labor was recorded and blood loss was measured during the first hour postpartum. For this latter purpose, blood was collected using specially adapted plastic bags. All these bags as well as the sponges and gazes used were then weighted and the blood loss (expressed in milliliters -ml) was measured by discounting the previously determined dry weight. After delivery, perineal conditions were evaluated by visually inspecting the vulvar introitus and perineal region. Whenever present, lacerations were classified as first degree (involving the skin and/or vaginal mucosa), second degree (affecting the perineal muscle), third degree (affecting the anal sphincter muscle) and fourth degree (in addition to the sphincter, the rectal mucosa is also affected) [7]. Third and fourth degree tears were classified as severe perineal damage.

The umbilical cord blood pH was measured in a sample collected from the fetal end of the cord after it was cut, with pH values ≥7.2 being considered normal.

Perineal pain was evaluated between 24 and 48 h following delivery by asking the woman if she had any discomfort in the perineal region. A visual analogue scale was used to estimate pain intensity, assessed as a score that ranged from 0 to 10, with zero indicating a complete absence of pain and 10 reflecting the worst pain imaginable [10, 11]. This variable was later recoded as “any perineal pain” (scores of 1 to 10). Next, maternal satisfaction was evaluated and classified as very satisfied, satisfied, fairly satisfied, dissatisfied or very dissatisfied in accordance with the woman’s selection from a range of faces in a faces scale [9].

The presence of complications with perineal suturing (edema, ecchymosis, hyperemia, secretion, hematoma, dehiscence, perineal pain and infection) was evaluated during hospitalization (until 48 h of delivery) and at the postpartum appointment scheduled 10 days after of vaginal delivery. Women were told they could return to the hospital before that date to the hospital if they felt it was necessary for suspecting any complications. At these times all the women had the perineal region routinely examined by a health professional (certified nurse-midwife or an obstetrician doctor) blinded to the group assignement.

A statistician blinded to the group assignment conducted the data analysis using the public domain Epi Info statistical software program (Atlanta, GA), version 7.2. The intention-to-treat principle was applied, i.e. each woman was analysed in the group to which she had originally been allocated. Student’s *t*-test or the Mann–Whitney test was used, as appropriate, for the numerical variables. The Pearson’s chi-square test or Fisher’s exact test (when one of the expected values was <5) was used for the categorical variables. A significance level of 5% was adopted. All *p*-values were two-tailed. Risk ratios were calculated as measures of relative risk (RR), together with their 95% confidence intervals (95% CI). A standard value of 1.0 was attributed to the reference category.

### Ethics, consent and permissions

The institution’s internal review board (Comitê de Ética em Pesquisa do IMIP) approved the study protocol under reference number 06561712.8.00005201. Eligible women were invited to participate in the study and they only were included if agreed voluntarily and signed an informed consent form.

## Results

**Fig. 1:**
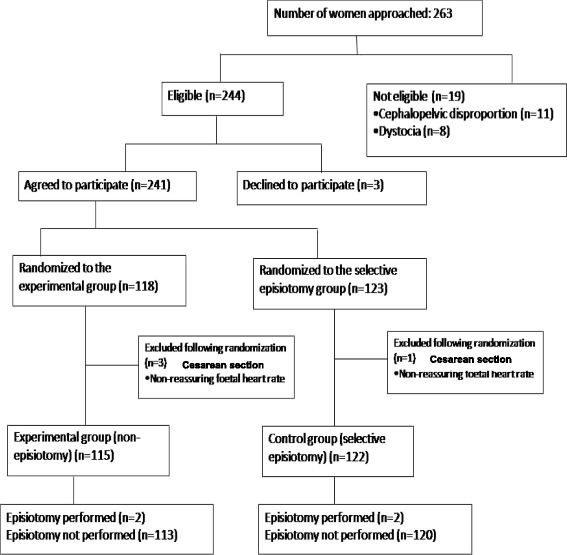
CONSORT flowchart

During the study period, 263 women in labor were considered for inclusion. Of these, 19 women were excluded (11 because there was cephalopelvic disproportion and 8 because of dystocia) and three women declined to participate. Therefore, 241 women were randomized, 118 to the non-episiotomy group and 123 to the selective episiotomy group. Four women were excluded following randomization because non-reassuring fetal heart rate developed and a caesarean section was indicated, leaving 115 women in the non-episiotomy group and 122 in the selective episiotomy group (Fig. 1).

The baseline characteristics of the women in the two groups were similar. Mean age was around 23 years, with a median number of pregnancies and live births of 1.5 and zero, respectively. The percentage of primiparous women in the study was 59.9%, with no statistically significant difference between the groups. The majority of the participants had nine or more years of formal schooling (around 70% of those included in the non-episiotomy group and 78% of those in the selective episiotomy group). Median gestational age at delivery was 39 weeks in both groups. The mean birthweight was similar in the two groups (3,284 g in the experimental group vs 3,260 g in the control group) (Table 1).

**Table 1:** Characteristics at baseline

Characteristic	Non-Episiotomy (*n* = 115)	Selective Episiotomy (*n* = 122)	*p*-value
Mother’s age			
Mean ± SD	23.9 ± 6.3	23.5 ± 5.6	0.60
Number of pregnancies			
Median (IQR)	1.5 (1.5 to 2.5)	1.5 (1.5 to 2.0)	0.59
Parity			
Median (IQR)	0.0 (0.0 to 1.0)	0 (0.0 to 1.5)	0.59
Education			
<9 years of schooling	35 (30.4%)	27 (22.1%)	0.14
≥ 9 years of schooling	80 (69.6%)	95 (77.9%)	
Gestational age at delivery (weeks)			
Median (IQR)	39 (38 to 40.5)	39 (38.0 to 39.0)	0.41
Birthweight			
Mean ± SD	3283.7 ± 408.1	3259.8 ± 398.4	0.68
<2500 g	02 (1.8%)	06 (4.9%)	0.75
2500 to 3999 g	107 (93.0%)	114 (93.4%)	
≥ 4000 g	06 (5.2%)	02 (1.7%)	

No significant differences were found in relation to the primary maternal outcomes evaluated (Table 2). The frequency of episiotomy was similar, 1.7% in the non-episiotomy group and 1.6% in the selective episiotomy group. The two episiotomies performed in the non-episiotomy group were indicated because of a prolonged second stage (35 and 120 min, respectively) in association with a non-reassuring fetal heart rate, while in the selective episiotomy group two episiotomies were performed exclusively due to a prolonged second stage (27 and 56 min, respectively). There was no significant difference between the experimental and control groups with respect to the duration of the second stage. The frequency of spontaneous lacerations was around 83% in both groups. There was only one case of an instrumental delivery (forceps) in the non-episiotomy group. Severe perineal trauma occurred in 1.8% of the women in the non-episiotomy group and in 2.5% of those randomized to the selective episiotomy group. There was also no difference in the mean postpartum blood loss between the experimental and control groups (257 ml versus 244 ml, respectively). Around 77% of the women in both groups required suturing. There was no difference in the number of suture threads required, with a median of around 1.7 threads in both groups.

**Table 2:** Non-episiotomy versus selective episiotomy: primary maternal outcomes

Variable	Non-Episiotomy		Selective Episiotomy		RR	95%CI	*p*-value
	N	%	N	%			
Duration of 2nd stage							
Mean ± SD (min)	27.2 ± 32.2		23.5 ± 21.7				0.8*
>1 h	09	7.8	06	4.9	1.59	0.58 to 4.33	0.36
≤ 1 h	106	92.2	116	97.5			
Episiotomy							
Yes	02	1.7	02	1.6	1.03	0.38 to 2.76	0.999*
No	113	98.3	120	98.4			
Spontaneous laceration							
Yes	95	82.6	102	83.6	0.96	0.68 to 1.35	0.86*
No	20	17.4	20	16.4			
Instrumental delivery							
Yes	01	0.8	0	-	NC	-	0.97*
No	114	99.2	122	100.0			
Severe perineal trauma							
Yes	02	1.8	03	2.5	0.85	0.41 to 1.76	0.999*
No	113	98.2	119	97.5			
Postpartum blood loss							
Mean ± SD	257.4 ± 200.4		244.5 ± 314.8				0.41
>500 ml	16	13.9	09	7.4	1.89	0.87 to 5.0	0.10
≤ 500 ml	99	86.1	113	92.6			
Need for perineal suturing							
Yes	89	77.4	94	77.1	1.00	0.75 to 1.35	0.999*
No	26	22.6	28	22.9			
Number of suture threads							
Median (IQR)	1.65 (1.0 to 2.0)		1.76 (1.0 to 2.0)				0.54**
≥ 2 threads	51	52.1	57	55.8	0.93	0.70 to 1.22	0.67*
1 thread	47	47.9	45	44.2			

*RR* relative risk, *CI* confidence interval, *IQR* Interquartile range, *NC* Not calculated

*Fisher two-tailed test; **Mann–Whitney test due to different variants or discrete variables

The groups were not significantly different insofar as any of the primary perinatal outcomes were concerned either. Apgar scores were similar, with only two cases of 5^th^ minute Apgar scores <7 in the selective episiotomy group. Neonatal resuscitation was required in around 3% of cases in each group and there was only one case of umbilical cord blood acidosis in the selective episiotomy group (Table 3).

**Table 3:** Non-episiotomy versus selective episiotomy: primary perinatal outcomes

Variable	Non-Episiotomy		Selective Episiotomy		RR	95%CI	*p*-value
	N	%	N	%			
1^st^ minute Apgar score							
<7	07	6.1	10	8.2	0.74	0.29 to 1.89	0.53
≥ 7	108	93.9	112	91.8			
5^th^ minute Apgar score							
<7	0	-	02	1.7	NE	-	0.53*
≥ 7	115	100.0	120	91.8			
Neonatal resuscitation							
Yes	03	2.6	04	3.3	0.89	0.47 to 1.72	>0.999*
No	112	97.4	118	96.7			
Umbilical cord blood acidosis							
Yes	0	-	1	0.8	NE	-	>0.999*
No	115	100.0	121	99.2			

*RR* relative risk, *CI* confidence interval

*Fisher’s two-tailed exact test

Analysis of the secondary maternal outcomes showed no significant difference between groups. The frequency of postpartum perineal pain was similar, around 50%, in both groups. There were no cases of dehiscence, hematoma or wound infection in either of the groups. Around 99% of the women in the non-episiotomy group were satisfied or very satisfied compared to 96% in the selective episiotomy group, with no significant difference between the groups (Table 4).

**Table 4:** Non-episiotomy versus selective episiotomy: secondary maternal outcomes

Variable	Non-Episiotomy		Selective Episiotomy		RR	95% CI	*p*-value
	n	%	n	%			
Wound dehiscence							
Yes	-	-	-	-	-	-	-
No	-	-	-	-	-	-	-
Wound hematoma							
Yes	-	-	-	-	-	-	-
No	-	-	-	-	-	-	-
Wound infection							
Yes	-	-	-	-	-	-	-
No	-	-	-	-	-	-	-
Postpartum perineal pain							
Yes	59	51.3	60	49.2	1.04	0.81 to 1.33	0.79
No	56	48.7	62	50.8			
Maternal satisfaction							
Satisfied/very satisfied	114	99.1	117	95.9	1.03	0.99 to 1.08	0.24*
Dissatisfied/very dissatisfied	01	0.9	05	4.1			

*RR* relative risk, *CI* confidence interval

*Fisher’s two-tailed exact test

There were no significant differences between the groups with respect to the secondary perinatal outcomes either, with around 3% of the newborn infants in both groups requiring some form of oxygen therapy and 3% requiring admission to the neonatal ICU. The duration of neonatal hospitalization was also similar in the two groups, with only 0.9% of those in the non-episiotomy group and 1.6% of those in the selective episiotomy group needing to stay in hospital for 48 h or more (Table 5).

**Table 5:** Non-episiotomy versus selective episiotomy: secondary perinatal outcomes

Variable	Non-episiotomy		Selective episiotomy		RR	95%CI	*p*-value
	N	%	N	%			
Oxygen therapy							
Yes	03	2.6	04	3.3	0.89	0.47 to 1.73	>0.999*
No	112	97.4	118	96.7			
Admission to neonatal ICU							
Yes	03	2.6	03	2.5	1.03	0.45 to 2.31	>0.999*
No	112	97.4	119	97.5			
Duration of neonatal hospitalization							
≥ 48 h	01	0.9	02	1.6	0.5	0.08 to 2.99	>0.999*
<48 h	02	1.7	01	0.8			

*RR* relative risk, *CI* confidence interval, *ICU* intensive care unit

*Fisher’s two-tailed exact test

## Discussion

### Principal findings

In the present study-, no difference was found between the women randomized to the selective episiotomy group compared to those randomized to the non-episiotomy group. The overall rate of episiotomy was very low (around 1.7%), similar in the two groups, and close to the low rates already described by other authors [9, 12]. The episiotomy rate found in the present study is well below the maximum of 10% recommended by the WHO [3], and much lower than the overall episiotomy rate found in a Cochrane systematic review of around 28% in the group submitted to selective episiotomy [5].

### Strengths and weaknesses

Episiotomy was introduced into obstetric practice without any scientific evidence corroborating any possible benefits [13]. Its use became widespread in the twentieth century based on the recommendation of renowned obstetricians such as Gabbe and DeLee [14]. The paradigm that existed at that time was interventionist to the extreme in that the female body was believed to be essentially defective and dependent on medical interventions to enable childbirth to take place [15]. This belief, together with a change in the place of childbirth, away from the home and into the hospital environment, contributed to the popularization of the procedure in hospitals [13]. It was only in the 1970s that discordant voices began to be heard, generally from within women’s movements, demanding changes in the obstetric model [13].

The review published by Thacker and Banta in 1983 not only highlighted the lack of any scientific studies supporting the use of episiotomy, but also found the practice to be potentially associated with harmful consequences such as perineal pain, hematoma, infection, dyspareunia and healing complications [16]. In 1984, the results of the first randomized clinical trial on the subject were published. That study, conducted in the United Kingdom, reported an episiotomy rate of 10% when the proposal was to perform the procedure selectively [4]. Various other randomized clinical trials followed and are summarized in a Cochrane systematic review. The well-documented advantages of restricting the practice of episiotomy rather than encouraging its routine use include less risk of posterior perineal trauma and of severe perineal trauma. Other positive consequences are less blood loss, less need for sutures, a lower frequency of postpartum perineal pain, a lower risk of perineal suture complications (oedema, dehiscence, infection and hematoma), fewer cases of postpartum loss of perineal muscle strength and less risk of dyspareunia [5].

A question that has recently been raised is whether there is indeed any indication for performing episiotomy and whether the procedure, even when practiced selectively, confers any benefit at all, either immediately or later [1, 6, 9]. Indications such as a prolonged second stage, macrosomia, non-reassuring fetal heart rate, instrumental delivery, occiput posterior position, pelvic delivery and shoulder dystocia have been questioned [9]. A systematic review of the effectiveness of episiotomy for prevention and management of shoulder dystocia found no evidence supporting the use of episiotomy [17]. The American College of Obstetricians and Gynecologists recognizes that there is insufficient objective evidence-based criteria to define the indications for episiotomy – and that restrictive use of episiotomy remains the best practice [2].

### Strengths and weaknesses in relation to other studies

In one study that included 168,077 vaginal births at the University of Soroka Medical Centre, Israel, mediolateral episiotomy was found to be an independent risk factor for third and fourth degree perineal lacerations, even in critical situations such as shoulder dystocia, instrumental deliveries, posterior presentations, fetal macrosomia and non-reassuring fetal heart rate. The episiotomy rates at that hospital fell from over 30% in the 1990s to less than 5% in 2010 [12].

Using a non-episiotomy protocol associated with strategies aimed at protecting the perineum, Amorim et al. reported an intact perineum rate of approximately 60%, with only 23% of women requiring perineal suturing [9, 18]. That study, however, was not controlled and was conducted with a specific sample of women, and the authors recommended that randomized clinical trials should be performed to compare a non-episiotomy policy with one of selective episiotomy. However, other observational studies corroborate that it is possible to avoid episiotomy and achieve a high rate of intact perineum. In other larger series of 1,176 women with natural births without episiotomy, an intact perineum rate of almost 65% was reported with 20% of perineal suture need [19]. Recently, a retrospective study conducted in Tokyo, Japan, including 1,1521 women with spontaneous births without interventions (epidural, episiotomy, instrumental delivery) reported intact perineum rates of 49.5% in nulliparous and 69.9% in multiparous women, with only 0.1% of third-degree laceration (one case) [20].

Notwithstanding, all above mentioned studies were observational, descriptive, uncontrolled studies with the limitations inherent to the study design and until this moment we did not identify any randomized controlled trial with the objective to determine if episiotomy is necessary in any circumstance. The current recommendation of selective episiotomy is based on the conclusions of systematic reviews of clinical trials that demonstrated selective episiotomy was better than routine episiotomy [5, 25] and the authors of the original clinical trials did not “dare” to address the question if it is possible to never perform episiotomies for vaginal deliveries, the results and safety of definitively abolishing this procedure of the modern obstetric practice.

The present study is the first randomized clinical trial to compare the restricted use of episiotomy with a non-episiotomy protocol, thus classifying its results as representing an original contribution to the current literature. The advantages of the study include its design, in which the women were randomly allocated to one of two groups, and the sample size, which was sufficient to show any possible benefits or harmful effects in either of the two groups. The analysis was performed on an intention-to-treat basis, which explains the two cases of episiotomy in the group of women randomized to the non-episiotomy group. Since the indications recorded for these episiotomies were a “prolonged second stage” associated with non-reassuring fetal heart rate, the procedure may have been avoidable. Likewise, in the selective episiotomy group, the two episiotomies performed because of a “prolonged second stage” may have been unnecessary. The considered limits for the duration of the second stage are currently more flexible. If mother and baby are well, patience to wait without intervening could have avoided some of the procedures performed [21–23].

However, the present randomized clinical trial may have been performed too late at this hospital, where episiotomy rates have been falling for the past ten years, reaching a level below 2% within the context of selective practice in this study. This change in the paradigm could explain these results. Since this study was conducted at one single centre, further randomized clinical trials need to be carried out, preferably in places where selective episiotomy rates are higher in order to verify whether there really are any relevant differences when the procedure is performed, even with restricted indications, versus when there is no intention to ever perform the procedure. Ideally, a future systematic review could include randomized clinical trials conducted for this purpose to enable solid recommendations to be proposed for routine obstetric practice.

### Explanations and implications for clinicians and policymakers

Until the results of such studies are available, it appears reasonable to propose that the World Health Organization redefine its cut-off point for the “ideal” episiotomy rate and, in an effort to reduce rates, that nurses, midwives and doctors be trained not to perform the procedure indiscriminately.

Despite the consistent evidence against its indiscriminate practice [5], in some places episiotomy is still performed routinely and indeed a recent study published in Brazil showed a rate of 54% in this country [24]. This rate is more than five times the maximum rate recommended by the World Health Organization and means that a large number of healthcare professionals (particularly doctors, since in the current obstetric model in Brazil the majority of deliveries are performed by doctors) still carry out this procedure systematically. This may imply additional costs for the healthcare system, since, just in suture threads, savings between $6.50 and $12.50 could be made with each vaginal delivery. Carroli and Belizan evaluated that this could represent a current annual saving of US$ 15 to 30 million for Brazil [25].

Furthermore, routine episiotomy is now considered a form of obstetric violence, particularly when performed without informed consent. A relatively new legal term, “obstetric violence” is used to describe a situation in which any form of labor in childbirth is considered pathological, when a woman is automatically transformed into a patient and when routine medical and pharmacological procedures are carried out without giving the woman the right to make her own decisions with respect to her own body [26]. Routinely performing procedures that, in addition to being unnecessary, may be harmful, characterizes mistreatment during childbirth [27]. In this context, routine or high rates of episiotomy can be categorized as female genital mutilation [28].

### Unanswered questions and future research

Within this context, it is important to define whether episiotomy is really necessary in any situation and what, if any, are its true indications. In addition to the harmful effects that may be associated with episiotomy and the costs of the procedure to the healthcare system, this question also deals with issues of reproductive rights.

It may seem curious that a procedure would be incorporated into obstetric practice without a shred of evidence of its beneficial effects and our task now is to generate evidence to determine whether its use is in fact necessary at any childbirth. This evidence should have been sought in the past. As Cochrane said: “*One should…always assume that a treatment is ineffective unless there is evidence to the contrary*” [29]. Unfortunately, the randomized clinical trials that were published following publication of the paper by Thacker and Banta did not compare the non-performance of episiotomy with the routine practice of this procedure [30, 31].

It is hoped that this study may contribute towards significantly reducing episiotomy rates in Brazil and in other places where routine episiotomy is still performed. According to a recent study comparing rates of episiotomy in 20 European countries found important differences, varying from 3,7% in Denmark to 75% in Cyprus [32]. In other institutes and in other countries in which episiotomy is practiced selectively, the present results are expected to encourage further studies to find answers to the more decisive questions: Is it time to stop performing episiotomies? Have we actually reached the point of proposing to abolish episiotomy? It is our belief that, yes, this is the end of the cut.

## Conclusions

An overall episiotomy rate of less than 2% was found in the two groups evaluated in the present study, showing that it is possible to implement a non-episiotomy protocol for a group of women with full-term pregnancies and foetuses in the cephalic position without any statistically significant differences in relation to the selective practice of the procedure. This is the first randomized clinical trial comparing the intention to never perform episiotomy to the current recommended practice of selective episiotomy. Further studies should be conducted, particularly in institutions and regions in which episiotomy rates remain high (although under the concept of “restrictive” use), in an attempt to determine whether there really is any indication for episiotomy in modern obstetrics. Until the results of these studies are available, much lower episiotomy rates should be achieved in this country and in other countries, and the end of episiotomy is a goal that should be pursued within a humanized childbirth care model.

## References

[1] Melo I, Katz L, Coutinho I, Amorim MM (2014). Selective episiotomy vs. implementation of a non episiotomy protocol: a randomized clinical trial. Reprod Health.

[2] American College of Obstetricians-Gynecologists (2016). ACOG Practice Bulletin. Practice Bulletin No. 165: Prevention and Management of Obstetric Lacerations at Vaginal Delivery. April 2016. Obstet Gynecol.

[3] World Health Organization Division of Family Health Maternal Health and Safe Motherhood. Care in normal birth: a practical guide. Report of a technical working group. World Health Organisation, 1996. http://apps.who.int/iris/bitstream/10665/63167/1/WHO_FRH_MSM_96.24.pdf.

[4] Sleep J, Grant A, Garcia J, Elbourne D, Spencer J, Chalmers I (1984). West Berkshire perineal management trial. Br Med J.

[5] Carroli G, Mignini L. Episiotomy for vaginal birth. Cochrane Database Syst Rev. 2009;(1):CD000081. 10.1002/14651858.CD000081.pub2 PMC4175536 19160176

[6] Lappen JR, Gosset DR (2010). Changes in episiotomy practice: evidence-based medicine in action. Expert Rev Obstet Gynecol.

[7] Scott JR (2005). Episiotomy and vaginal trauma. Obstet Gynecol Clin North Am.

[8] Eason E, Feldman P (2000). Much ado about a little cut: is episiotomy worthwhile?. Obstet Gynecol.

[9] Amorim MM, Franca-Neto A, Leal NV, Melo FO, Maia SB, Alves JN (2014). Is it possible to never perform episiotomy during vaginal delivery?. Obstet Gynecol.

[10] Chapman CR, Syrjala KL, Bonica JJ (1990). Pain. Lea & Febiger.

[11] Hicks CL, von Baeyer CL, Spafford PA, van Korlaar I, Goodenough B (2001). The Faces Pain Scale-Revised: toward a common metric in pediatric pain measurement. Pain.

[12] Steiner N, Weintraub AY, Wiznitzer A, Sergienko R, Sheiner E (2012). Episiotomy: the final cut?. Arch Gynecol Obstet.

[13] Myers-Helfgott MG, Helfgott AW (1999). Routine use of episiotomy in modern obstetrics. Should it be performed?. Obstet Gynecol Clin North Am.

[14] Gabbe SG, DeLee JB (2002). The prophylactic forceps operation. 1920. Am J Obstet Gynecol.

[15] Davis-Floyd R. Birth as an American rite of passage. Berkeley: University of California Press; 1993:382.

[16] Thacker SB, Banta HD (1983). Benefits and risks of episiotomy: an interpretative review of the English language literature, 1860–1980. Obstet Gynecol Surv.

[17] Sagi-Dain L, Sagi S (2015). The role of episiotomy in prevention and management of shoulder dystocia: a systematic review. Obstet Gynecol Surv.

[18] Leal NV, Amorim MM, Franca-Neto AH, Leite DF, Melo FO, Alves JN (2014). Factors associated with perineal lacerations requiring suture in vaginal births without episiotomy. Obstet Gynecol.

[19] Albers LL, Sedler KD, Bedrick EJ, Teaf D, Peralta P (2006). Factors related to genital tract trauma in normal spontaneous vaginal births. Birth.

[20] Suto M, Takehara K, Misago C, Matsui M (2015). Prevalence of Perineal Lacerations in Women Giving Birth at Midwife-Led Birth Centers in Japan: A Retrospective Descriptive Study. J Midwifery Womens Health.

[21] Zhang J, Landy HJ, Branch DW, Burkman R, Haberman S, Gregory KD (2010). Consortium on Safe Labor. Contemporary patterns of spontaneous labor with normal neonatal outcomes. Obstet Gynecol.

[22] El-Sayed YY (2012). Diagnosis and management of arrest disorders: duration to wait. Semin Perinatol.

[23] Caughey AB, Cahill AG, Guise JM, Rouse DJ, American College of Obstetricians and Gynecologists (College), Society for Maternal-Fetal Medicine (2014). Safe prevention of the primary cesarean section. Am J Obstet Gynecol.

[24] Carmo Leal M, Pereira AP, Domingues RM (2014). Obstetric interventions during labor and childbirth in Brazilian low-risk women. Cad Saude Publica.

[25] Carroli G, Belizan J (2000). Episiotomy for vaginal birth. Cochrane Database Syst Rev.

[26] Pérez D’Gregorio R (2010). Obstetric violence: a new legal term introduced in Venezuela. Int J Gynaecol Obstet.

[27] Bohren MA, Vogel JP, Hunter EC, Lutsiv O, Makh SH, Souza JP (2015). The mistreatment of women during childbirth in health facilities globally: a mixed-methods systematic review. PLoS Med.

[28] Belizán JM, Miller S, Salaria N (2016). We need to stop female genital mutilation!. Reprod Health.

[29] Cochrane AL. Effectiveness and efficiency: random reflections on health services. London: Nuffield Provincial Hospitals Trust; 1972.

[30] Routine vs selective episiotomy: a randomised controlled trial (1993). Argentine Episiotomy Trial Collaborative Group. Lancet.

[31] Thorp JM, Bowes WA, Brame RG, Cefalo R (1987). Selected use of midline episiotomy: effect on perineal trauma. Obstet Gynecol.

[32] Blondel B, Alexander S, Bjarnadóttir RI, Gissler M, Langhoff-Roos J, Novak-Antolič Ž, Prunet C, Zhang WH, Hindori-Mohangoo AD, Zeitlin J, Euro-Peristat Scientific Committee (2016). Variations in rates of severe perineal tears and episiotomies in 20 European countries: a study based on routine national data in Euro-Peristat Project. Acta Obstet Gynecol Scand.

