# Correction to: Selective episiotomy vs. implementation of a non-episiotomy protocol: a randomized clinical trial

**DOI:** 10.1186/s12978-017-0399-x

**Published:** 2017-10-24

**Authors:** Melania M. Amorim, Isabela Cristina Coutinho, Inês Melo, Leila Katz

**Affiliations:** 1 Instituto de Medicina Integral Prof. Fernando Figueira, Women’s Healthcare Centre, Recife, Pernambuco 50070-550 Brazil; 2 Instituto Paraibano de Pesquisa Professor Joaquim Amorim Neto, Rua Neusa Borborema, 300, Santo Antônio, Campina Grande, 58406-120 Brazil

## Correction

Unfortunately, the original version of this article [1] contained an error. One of the author’s full names was not included properly. It is included here correctly. The name of the author is Melania M. Amorim.
